# Predator egg‐induced non‐consumptive effects suppress spider mite survival and reproductive performance

**DOI:** 10.1002/ps.70976

**Published:** 2026-06-03

**Authors:** Resona Simkhada, Jhaman Kundun, Svetla Sofkova‐Bobcheva, Xiong Z He

**Affiliations:** ^1^ School of Agriculture and Environment Massey University Palmerston North New Zealand; ^2^ Nepal Agricultural Research Council Kathmandu Nepal

**Keywords:** biological control, life‐history strategy, *Phytoseiulus persimilis*, population growth, predation stress, *Tetranychus ludeni*

## Abstract

**BACKGROUND:**

Predators suppress pest populations not only through direct consumption but also via non‐consumptive effects (NCEs), in which prey perceive predation risk and alter behaviors, physiology, and life‐history strategies. Despite the growing recognition of the importance of NCEs in biological control, most studies focus on cues from actively feeding predators, while the role of non‐feeding stages, such as predator eggs, remains poorly understood. Here, we investigated whether eggs of the predatory mite *Phytoseiulus persimilis* function as intensity‐dependent risk cues that influence the performance and population growth of the invasive spider mite *Tetranychus ludeni*.

**RESULTS:**

Exposure to *P. persimilis* eggs significantly altered *T. ludeni* life‐history traits in a density‐dependent manner, even in the absence of direct predation. Increasing predator egg density reduced female longevity, reproductive rate, and total fecundity, delayed reproductive onset, but accelerated early egg production. Exposure to predator eggs also generated pronounced transgenerational effects that prolonged egg and female offspring development, reduced egg hatching, and lowered immature survival. These individual‐level responses translated into strong population‐level consequences, which significantly decreased the net reproductive rate (*R*_*0*_), intrinsic rate of increase (*r*_*m*_), and finite rate of increase (*λ*), but increased population doubling time (*Dt*) as predation stress intensified.

**CONCLUSION:**

Our results demonstrate that predator eggs induce intensity‐dependent NCEs that significantly suppress spider mite population growth. By extending fear‐mediated effects from non‐preying predator stages, this study broadens the functional scope of biological control and highlights the ecological significance of early predator cues in regulating pest populations. © 2026 The Author(s). *Pest Management Science* published by John Wiley & Sons Ltd on behalf of Society of Chemical Industry.

## Graphical abstract

Increasing predation intensity of predatory mite significantly reduced spider mite fitness in terms of shorter mother longevity, lower mother fecundity, longer offspring development, lower offspring survival, and lower population growth.

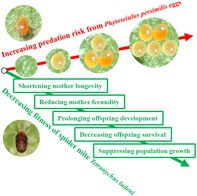

## 1 INTRODUCTION

Predators manage prey populations not only through direct consumption but also via non‐consumptive effects (NCEs), where the perception of predation risk alters prey behavior, physiology, and life‐history strategies.^1^, ^2^, ^3^, ^4^ Such risk‐induced responses can substantially suppress prey development and reproduction, often reducing population performance to an extent comparable to or even exceeding that caused by direct predation.^2^, ^5^, ^6^, ^7^, ^8^ In agricultural systems, NCEs are increasingly recognized as a critical but underutilized component of biological control,^9^, ^10^, ^11^, ^12^, ^13^ because predator presence alone can usually reduce pest damage by limiting feeding, slowing development, and constraining reproductive output.^2^, ^5^, ^10^, ^14^, ^15^, ^16^, ^17^

Prey rely on diverse sensory cues, including chemical, tactile, and visual signals, to assess predation risk and adjust their behaviors and physiology accordingly.^18^, ^19^, ^20^, ^21^, ^22^ Among these, chemical cues are particularly important in mediating NCEs across taxa, as they persist in the environment and convey information about predator identity, proximity, and recent feeding history.^22^, ^23^, ^24^, ^25^, ^26^, ^27^ Prey responses to predator‐derived cues are typically graded rather than binary (i.e., presence *vs*. absence), scaling with cue concentration or intensity and allowing prey to estimate the magnitude and immediacy of threat.^17^, ^21^, ^23^, ^28^, ^29^, ^30^ Stronger chemical cues may consistently elicit more pronounced antipredator responses, demonstrating that behavioral adjustments scale with cue intensity^21^ and animals are capable of integrating environmental information to evaluate threat levels.^31^, ^32^ However, such cue‐dependent risk assessment may induce trade‐offs between predator avoidance and investment in growth, reproduction, and survivorship, imposing substantial physiological and demographic costs.^12^, ^14^, ^17^, ^33^, ^34^

Spider mites in the genus *Tetranychus* (Acari: Tetranychidae) are among the most destructive arthropod pests in agricultural and horticultural systems worldwide, causing substantial crop losses due to their high fecundity, short generation time, rapid population growth, and frequent evolution of acaricide resistance.^35^, ^36^, ^37^, ^38^ Among various *Tetranychus spp*., the cosmopolitan invasive spider mite *Tetranychus ludeni* Zacher is an increasingly important agricultural pest, widely distributed in tropical and subtropical regions and capable of year‐round reproduction due to the absence of diapause.^39^, ^40^ Its high fecundity, short generation time, and ability to disrupt host‐plant physiology make it a persistent threat in agroecosystems.^41^, ^42^, ^43^ Biological control using predatory mites, particularly phytoseiid species such as *Phytoseiulus persimilis* Athias‐Henriot (Acari: Phytoseiidae) and *Neoseiulus californicus* McGregor (Acari: Phytoseiidae), forms a cornerstone of integrated pest management against spider mites.^44^, ^45^ Although *Tetranychus spp*. may readily evolve resistance to acaricides,^36^, ^46^, ^47^ *T. ludeni* remains highly susceptible to predation by *P. persimilis*, highlighting the importance of biological control for sustainable management.^39^

*Tetranychus* mites are capable of detecting and responding to predator cues through changes in behavior, reproduction, and habitat use.^12^, ^16^, ^17^, ^24^, ^30^, ^48^, ^49^, ^50^, ^51^, ^52^ While previous studies have mostly focused on the consumptive impacts of predatory mites on spider mite populations through investigating the prey preference and functional responses,^53^, ^54^, ^55^, ^56^, ^57^, ^58^, ^59^ or have focused on cues associated with actively feeding stages,^16^, ^52^, ^60^, ^61^ overlooking the possibility that non‐feeding stages may also convey ecologically relevant risk information. Predator eggs, in particular, may function as early indicators of imminent predation, as they signal forthcoming emergence of mobile juvenile predators, and may release chemical or contact cues detectable by prey.^62^, ^63^ So far, whether spider mites perceive predator eggs as reliable cues of future predation risk, and whether prey responses scale with egg density, remains poorly understood.

In the current study, we investigated whether eggs of the predatory mite *Phytoseiulus persimilis* act as intensity‐dependent non‐consumptive stressors that alter the life‐history traits and population performance of *T. ludeni*. By exposing spider mites to increasing densities of predator eggs while excluding direct predation, we tested whether predator egg cues (i) function as reliable indicators of predation risk, (ii) induce graded changes in survival and reproductive scheduling of adult females, (iii) generate transgenerational effects on offspring development and survival, and (iv) ultimately suppress population growth. This study linked egg cue intensity to individual‐ and population‐level responses, addressed a critical gap in spider mite ecology, and highlighted the potential for fear‐mediated effects from non‐feeding predator stages to enhance biological control in agricultural systems.

## 2 MATERIALS AND METHODS

### 2.1 Mite colonies and environmental conditions

The colony of *T. ludeni* was obtained from a home garden in Palmerston North, New Zealand, and reared and maintained on bean plants *Phaseolus vulgaris* L. (Fabales: Fabaceae) in the laboratory. The predatory mite *P. persimilis* was obtained from BioForce Limited, Auckland and reared on two‐spotted spider mite *T. urticae* on bean plants. These two colonies were kept in two separate bioassay rooms at 25 ± 1°C, and 14:10 h (light: dark) photoperiod, with a 40 ± 10% RH for *T. ludeni* and a 65 ± 10% RH for *P. persimilis*.

### 2.2 Experiment, data collection, and data calculation

Four treatments were established to quantify the effect of predation intensity on reproductive performances of *T. ludeni*: (i) high predation intensity – a leaf arena of 2 cm^2^ with five predator eggs, (ii) moderate predation intensity – a leaf arena with three predator eggs, (iii) low predation intensity – a leaf arena with one predator egg, and (iv) no predation risk (control) – a leaf arena without any predator egg. There were 15 replicates for each treatment. To obtain predator eggs for the experiment, 30 mated females of *P. persimilis* were randomly collected from the colony and individually introduced onto a leaf square (4 cm × 4 cm) infected with 20 *T. ludeni* eggs and placed upside down on a water‐saturated cotton pad in a Petri dish (9 cm diameter × 1 cm height). After laying eggs for 24 h, the predator was transferred to another leaf square as mentioned above. To prepare mated *T. ludeni*, quiescent female deutonymphs were randomly collected from the colony and individually paired with virgin males for 24 h to ensure that all females were mated. Virgin males developed from eggs laid by virgin females. Mated females that were one day old were selected for the experiments.

For each replicate, predator eggs of the desired number were carefully transferred into a leaf arena using a fine hairbrush. A mated *T. ludeni* female was then introduced onto the leaf arena to oviposit. After 24 h, *T. ludeni* female was transferred to a new leaf arena with the same predator egg density. The predator eggs were also removed from the leaf arena daily, and replaced with fresh eggs (< 24 h) prepared as mentioned above to maintain a constant egg age and density throughout the experimental period. The number of eggs laid by the *T. ludeni* female was daily recorded under a stereomicroscope (Leica MZ12, Germany). These procedures were repeated daily until the female died. We recorded the parameters, including the female longevity, pre‐oviposition period (from the initial of experiment to the first egg laid), oviposition period (from the first to last eggs laid), total fecundity, reproductive rate (total fecundity divided by longevity), developmental periods of eggs (from oviposition to egg hatching) and offspring (from oviposition to adult emergence), egg hatching rate (number of larvae divided by number of eggs), and immature survival rate (number of adults divided by number of larvae). Newly emerged adults were sexed and removed from the leaf arena daily.

### 2.3 Statistical analysis

We calculated the life table parameters using data on daily survival and daughter production of females.^64^ The intrinsic rate of increase (*r*_*m*_, daughters/female/day) was calculated by solving the Lotka‐Euler equation, $\sum e^{-r_{m}x}l_{x}m_{x}$= 1, where *x* is the pivotal age, $l_{x}$ is the proportion of females surviving to age *x*, and $m_{x}$ is the number of daughters produced per female at age *x*. Other life table parameters included the net reproductive rate (*R*_*0*_ = $\sum l_{x}m_{x}$, daughters/female/generation), finite rate of increase (*λ* = $e^{r_{m}}$), mean generation time [*T* = $\log_{e}\left(R_{0}\right)/r_{m}$, days], and doubling time [*Dt* = $\log_{e}\left(2\right)/r_{m}$, days]. The bootstrap method with 50 000 bootstrap samples^65^, ^66^ was used to calculate the pseudo‐values of a given parameter for each female in each treatment.

All data were analyzed using SAS software (SAS 9.4, SAS Institute Inc., Cary, NC). Rejection level of H_0_ was set at *P* < 0.05. Data on the female longevity, oviposition period, oviposition rate (eggs/day), and total fecundity after ln(*x*) transformation were normally distributed (Shapiro–Wilk test, UNIVARIATE Procedure) and thus analyzed using an analysis of variance (ANOVA, GLM procedure) with a Tukey's Studentized Range (HSD) Test for multiple comparisons between treatments. However, other data were not normally distributed, thus a generalized linear model with a gamma distribution and a log‐link function (GLIMMIX Procedure) was applied to analyze data on the pre‐oviposition period, offspring development, and life table parameters, and a logistic linear model (GENMOD Procedure) with a binomial distribution and logit‐link function was used to compare egg hatching and immature survival rates between treatments. An adjusted Tukey–Kramer test was performed for multiple comparisons. An asymptotic exponential model^67^ was applied to fit the daily cumulative number of eggs: Cumulative eggs = *a*/{1 + exp[−*b*(*Age*‐*Age*_0_)]}, where *a* is the maximum value of egg cumulation, *Age*_0_ is the inflection point at which the instantaneous growth rate is maximized, *b* is the constant growth rate of the cumulation curve. The difference in *b* or *Age*_0_ was compared between treatments,^68^ i.e., if the 95% confidence limits (95% CLs) overlap, then there is no significant difference.

## 3 RESULTS

### 3.1 Effects of predation stress on adult survival and female reproduction

**Figure 1:**
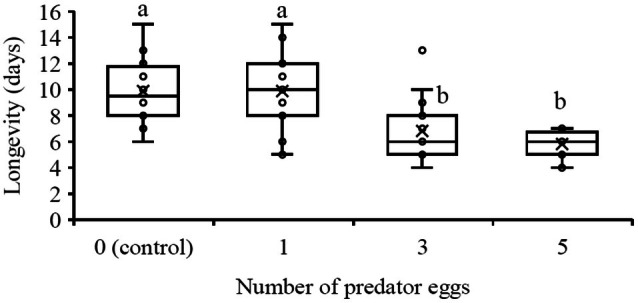
Longevity of *Tetranychus ludeni* females exposed to predation stress of different intensities. In each box plot, the line and ‘×’ in the box show the median and mean, respectively; the lower and upper box lines indicate the interquartile range [25th (Q1) to 75th (Q3) percentiles]; the lower and upper whiskers represent the scores outside Q1 and Q3, respectively, and the open circles (‘o’) present the individual data points. Boxes with different letters are significantly different (*P* < 0.05).

**Figure 2:**
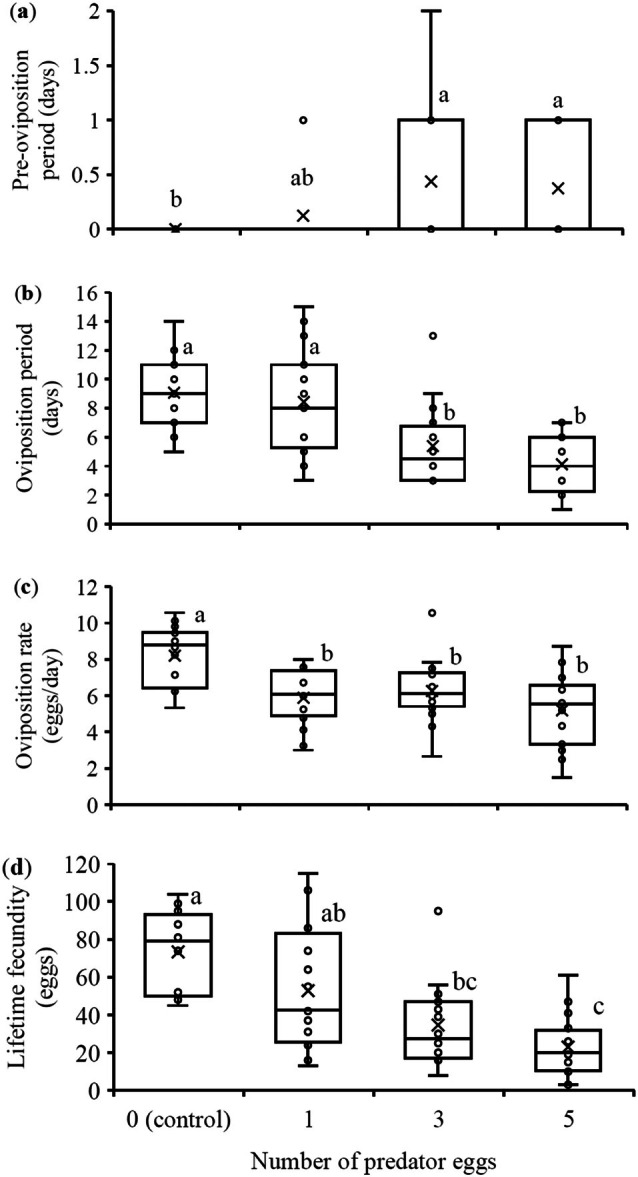
Pre‐oviposition period (a), oviposition period (b), reproductive rate (c), and lifetime fecundity (d) of individual *Tetranychus ludeni* females exposed to predation stress of various intensities. In each box plot, the line and ‘×’ in the box show the median and mean, respectively; the lower and upper box lines indicate the interquartile range [25th (Q1) to 75th (Q3) percentiles]; the lower and upper whiskers represent the scores outside Q1 and Q3, respectively, and the open circles (‘o’) present the individual data points. Boxes with different letters are significantly different (*P* < 0.05).

**Figure 3:**
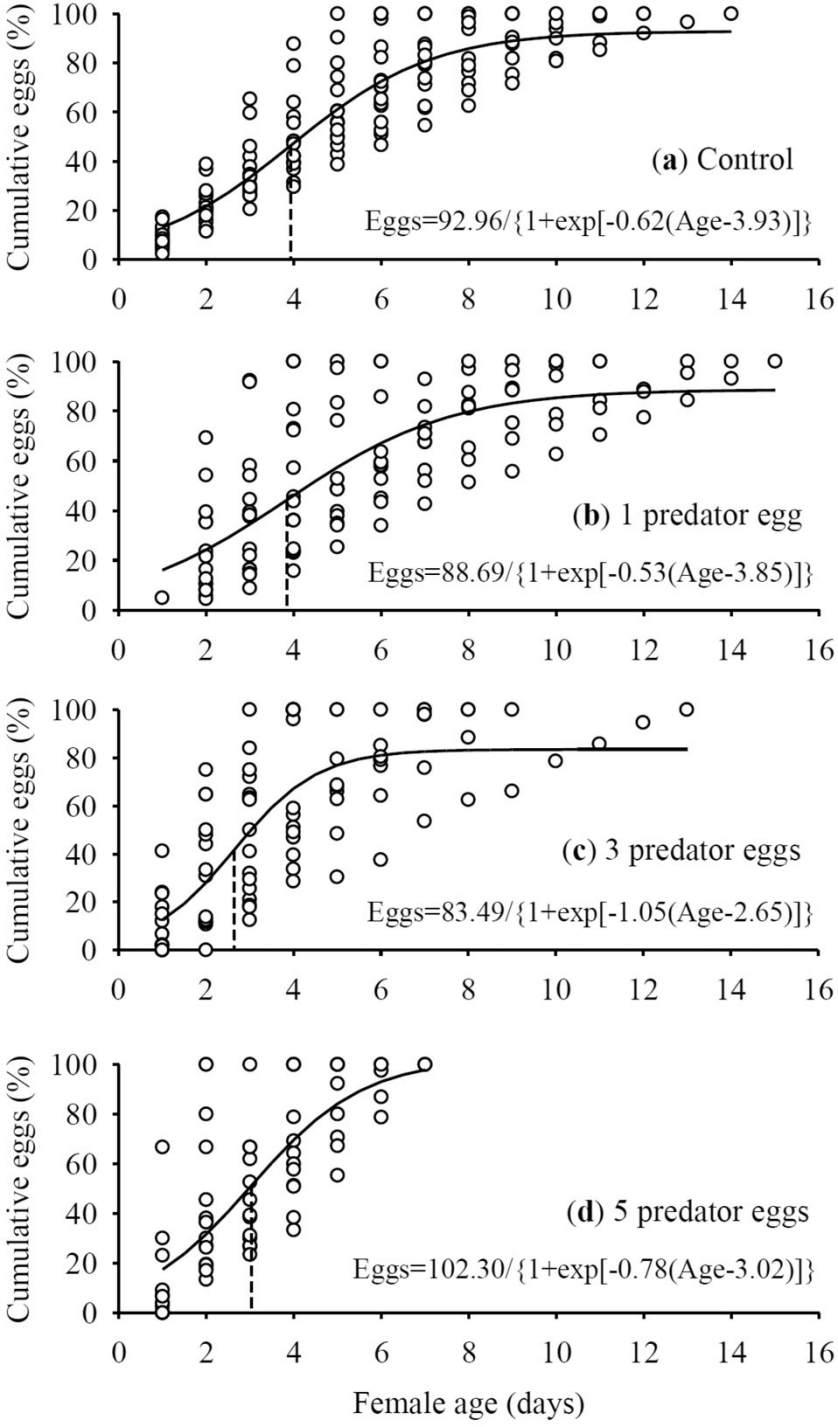
Proportion of egg cumulation over the reproductive period of *Tetranychus ludeni* females exposed to predation stress of various intensities: (a) Control, (b) one predator egg, (c) three predator eggs, and (d) five predator eggs. Solid line is the model prediction: Cumulative eggs = *a*/{1 + exp[−*b*(*Age*‐*Age*_0_)]}, where *a* is the maximum value of egg cumulation, *Age*_0_ is the inflection point at which the instantaneous growth rate is maximized (i.e., dash line), *b* is the constant growth rate of the cumulation curve. The open circles (‘o’) represent individual data points.

Females exposed to low predation stress (one predator egg) survived similarly with the control; however, those exposed to higher predation stress (i.e., three and five predator eggs) significantly shortened their longevity by 31.0% and 41.1%, respectively, compared to the control and low predation stress (*F*_3,60_ = 12.48, *P* < 0.0001) (Fig. 1). Females had a significantly longer per‐oviposition period when they were exposed to three or five predator eggs compared to the control (*F*_3,60_ = 5.64, *P* = 0.0018) (Fig. 2(a)), but a significantly shorter oviposition period compared to the control and that exposed to lower predation stress (i.e., 1 predator egg) (*F*_3,60_ = 11.50, *P* < 0.0001) (Fig. 2(b)). Exposure to predation stress regardless of the intensity significantly reduced the reproductive rate of females (*F*_3,60_ = 8.72, *P* < 0.0001) (Fig. 2(c)), and increasing predation stress significantly decreased the total fecundity of *T. ludeni* females by 27.9%, 52.8%, and 68.7%, respectively, compared to the control (*F*_3,60_ = 13.40, *P* < 0.0001) (Fig. 2(d)). Compared to the control, the proportion of egg cumulation was significantly faster when females were exposed to three predator eggs (nonoverlapping 95% CLs) and the schedule of maximum instantaneous growth rate was significantly early when females were exposed to three or five predator eggs (nonoverlapping 95% CLs) (Fig. 3).

### 3.2 Effects of predation stress on offspring fitness

**Figure 4:**
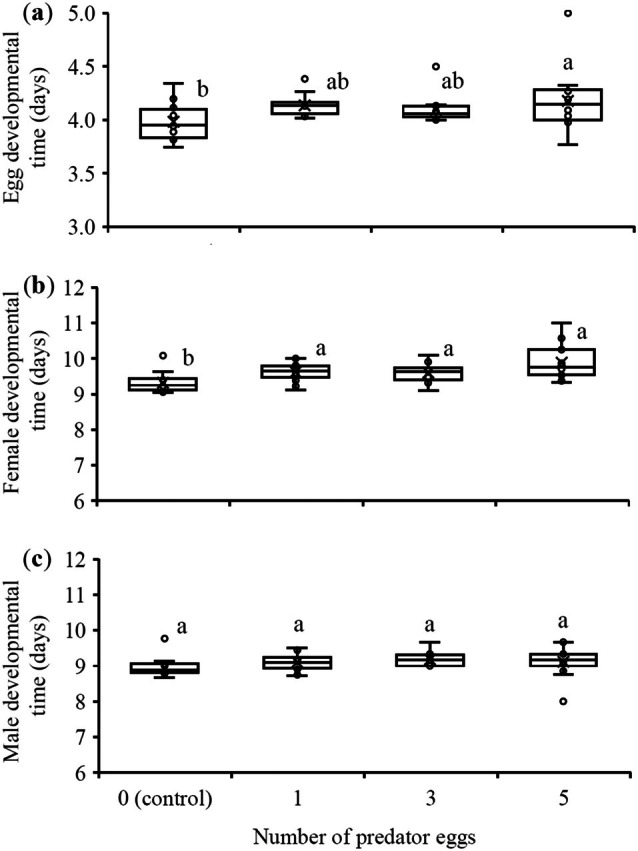
Developmental time of eggs (a), female offspring (b), and male offspring (c) of *Tetranychus ludeni* females exposed to predation stress of different intensities. In each box plot, the line and ‘×’ in the box show the median and mean, respectively; the lower and upper box lines indicate the interquartile range [25th (Q1) to 75th (Q3) percentiles]; the lower and upper whiskers represent the scores outside Q1 and Q3, respectively; and the open circles (‘o’) present the individual data points. Boxes with different letters are significantly different (*P* < 0.05).

**Figure 5:**
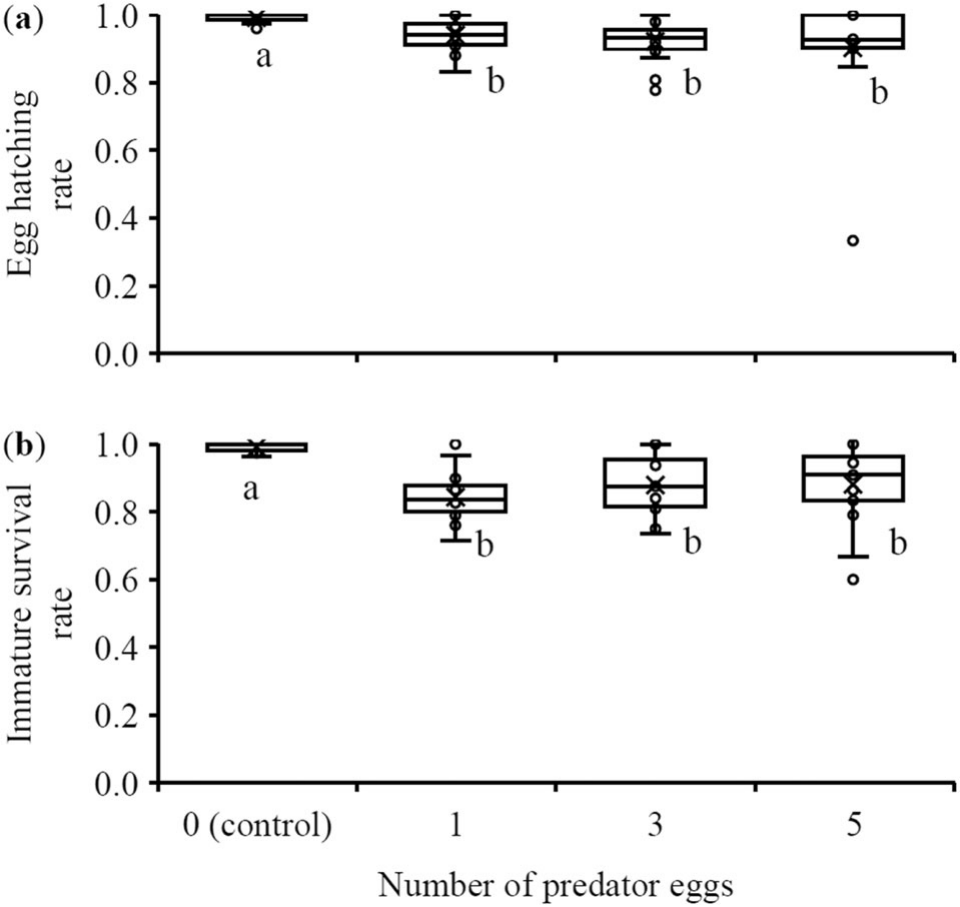
Egg hatching rate (a) and immature survival rate (b) when the ovipositing *Tetranychus ludeni* females were exposed to predation stress of different intensities. In each box plot, the line and ‘×’ in the box show the median and mean, respectively; the lower and upper box lines indicate the interquartile range [25th (Q1) to 75th (Q3) percentiles]; the lower and upper whiskers represent the scores outside Q1 and Q3, respectively, and the open circles (‘o’) present the individual data points. Boxes with different letters are significantly different (*P* < 0.05).

Eggs laid by females exposed to five predator eggs significantly prolonged development compared to the control eggs (*F*_3,60_ = 4.16, *P* = 0.0096) (Fig. 4(a)), and female offspring required significantly longer time to complete development from egg to adulthood when their mothers were exposed to any intensity of predation stress than the control (Fig. 4(b)) (*F*_3,59_ = 8.57, *P* < 0.0001); however, predation stress had no significant impact on development of male offspring (*F*_3,59_ = 2.00, *P* = 0.1243) (Fig. 4(c)). Predation stress significantly decreased egg hatch rate ($x_{3}^{2}$ = 84.42, *P* < 0.0001) (Fig. 5(a)) and immature survival rate ($x_{3}^{2}$ = 210.02, *P* < 0.0001) (Fig. 5(b)).

### 3.3 Effects of predation stress on population growth

Exposure to predation stress and increasing its intensity significantly decreased the net reproductive rate (*R*_*0*_), intrinsic rate of increase (*r*_*m*_), and finite rate of increase (*λ*), but significantly increased the doubling time (*Dt*) of *T. ludeni* population (Table 1). The generation time (*t*) was significantly longer under predation stress of one predator egg compared to other treatments, and it was significantly longer under control conditions than under the higher predation stress of five predator eggs (Table 1).

**Table 1:** Life table parameters of *Tetranychus ludeni* females exposed to predation stress (PS) of various intensities: net reproductive rate (*R*_*0*_), intrinsic rate of increase (*r*_*m*_), finite rate of increase (*λ*), generation time (*T),* and doubling time (*Dt*)

PS	*R*_*0*_ (daughters)	*r*_*m*_ (daughters/female/day)	*λ*	*T* (days)	*Dt* (days)
Control	52.37 ± 0.26 a	0.3227 ± 0.0001 a	1.3808 ± 0.0013 a	13.27 ± 0.03 b	2.14 ± 0.01 d
1 egg	41.49 ± 0.53 b	0.2722 ± 0.0008 b	1.3128 ± 0.0010 b	13.69 ± 0.05 a	2.55 ± 0.01 c
3 eggs	20.94 ± 0.22 c	0.2499 ± 0.0006 c	1.2839 ± 0.0008 c	12.17 ± 0.03 bc	2.77 ± 0.01 b
5 eggs	14.75 ± 0.19 d	0.2219 ± 0.0011 d	1.2485 ± 0.0015 d	12.12 ± 0.04 c	3.12 ± 0.02 a
*F*_3,60_	3072.38	2077.70	2393.00	443.25	2085.34
*P*	< 0.0001	< 0.0001	< 0.0001	< 0.0001	< 0.0001

For each parameter, means (± SE) followed by different letters are significantly different (Adjusted‐Tukey test: *P* < 0.05).

## 4 DISCUSSION

Our study demonstrates that predator‐derived cues, even from non‐feeding life stages, induced significant and intensity‐dependent stress responses in the spider mite *T. ludeni*. By quantifying the impacts of predator egg density on mite physiology and demography, we show that non‐consumptive effects (NCEs) fundamentally reshaped prey life‐history strategies, imposed transgenerational costs, and ultimately suppressed population growth. These findings highlight the importance of integrating risk perception, beyond direct predation, into our understanding of predator–prey dynamics and biological control efficacy.

### 4.1 Predator eggs function as reliable, intensity‐dependent cues of non‐consumptive predation stress

Results of the present study show that the presence of eggs of predatory mite *P. persimilis* significantly shortened *T. ludeni* longevity (Fig. 1), suppressed reproduction (Fig. 2), delayed egg and female development (Fig. 4(a), (b)), and decreased egg hatching and immature survival (Fig. 5), and such negative impacts on prey performances usually increased with the number of predatory eggs. These results may have significant implications. First, predator eggs may serve as reliable indicators of imminent predation threat, thereby providing early warning signals that allow prey to adjust life‐history strategies before direct encounter or consumption occurs.^62^, ^63^ It is well known that prey may detect predator‐borne cues including chemicals that dissipate from predators and their products, such as eggs, excreta, and pheromones (e.g., 24, 48, 50). In fact, spider mites could perceive and assess future predation risk even in the absence of active predators.^16^, ^52^, ^60^, ^61^, ^69^, ^70^ Second, predator eggs alone elicited strong NCEs on *T. ludeni*. Our finding directly addresses a key gap identified in previous studies on spider mites, which have largely focused on predator feeding stages.^16^, ^52^, ^60^, ^61^ Third, the strong prey responses proportional to the density of predator eggs indicate that as reported in other *Tetranychus* species,^16^, ^17^, ^30^, ^52^, ^71^ *T. ludeni* interpreted predator cues as a gradient of predation threat rather than a binary (presence *vs* absence) signal. Such early‐stage risk perception allows prey to integrate predator cue intensity to estimate threat proximity, magnitude, and severity^17^, ^21^, ^22^, ^23^, ^30^ before predators become mobile or consumptive.

### 4.2 Predation stress reshapes female life‐history strategies through accelerated but costly reproductive scheduling

Exposure to predator egg cues under moderate to high predation stress (i.e., three and five predator eggs) substantially induced significant changes in the reproductive scheduling of *T. ludeni* females, characterized by the early acceleration of egg accumulation (Fig. 3), even though the late onset of reproduction (Fig. 2(a)). These adjustments in reproductive trajectories likely represent stress‐induced reproductive compensation or terminal investment strategies, whereby individuals increase immediate reproductive effort when future survival or reproductive opportunities are uncertain.^29^, ^72^, ^73^, ^74^, ^75^ In arthropods, predator‐induced stress frequently triggers shifts toward earlier reproduction, reflecting an attempt to secure reproductive output before potential death.^17^, ^76^

However, despite this apparent compensation, accelerated reproduction was accompanied by substantial costs. Compared to the control, *T. ludeni* females exposed to moderate to high predation stress significantly shortened their longevity by >30% (Fig. 1) and oviposition period by >40% (Fig. 2(b)), and reduced the daily reproductive rate by >17% (Fig. 2(c)) and total fecundity by >50% (Fig. 2(d)), suggesting that predation stress induced sustained stress in prey and compromised their fitness.^6^ The earlier prey death and decreasing reproductive performances could be attributed to physiological and behavioral changes induced by NCEs, such as the energetic costs of risk avoidance,^77^, ^78^ decrease of foraging rate or food intake,^25^, ^79^, ^80^ and increase of oxidative damage.^33^ In addition, predator cues may affect prey through physiological pathways by inducing stress hormones,^3^, ^74^, ^81^ which divert their resource allocation to other physiological processes that may translate into lower survivorship^14^, ^17^, ^34^ and reproduction.^12^, ^17^, ^82^ The shortened survival and reduced reproduction are expected to minimize the damage of *T. ludeni* to the infested crops.^12^

### 4.3 Predator‐induced maternal stress induces transgenerational fitness costs

The legacy of maternal predation stress extended to the next generation, imposing fitness costs, thereby amplifying the population‐level impact of NCEs. The delayed egg development (Fig. 4(a)), and prolonged development of female offspring (Fig. 4(b)) and reduced survival of eggs and immatures (Fig. 5) across all stress levels provided strong evidence for transgenerational phenotypic programming. Maternal stress alters offspring phenotype via several mechanisms, including changes in yolk hormone (e.g., ecdysteroid) or nutrient provisioning, deposition of maternal mRNAs, or epigenetic modifications.^83^, ^84^, ^85^ This prolonged development was likely due to the maternal stress transmission, as even short‐term gestational stress of the mother can override or interact with previous experiences to shape offspring performance.^74^, ^84^, ^86^ These transgenerational effects ensure that the risk impact of predator cues persists well beyond the lifespan of the initially exposed individuals.

Interestingly, immature developmental delay in *T. ludeni* under predation stress is sex‐specific, affecting female but not male offspring (Fig. 4(b), (c)). This pattern suggests a differential sensitivity or investment between sexes, with female offspring bearing greater costs of maternal stress. Sex‐specific responses to predation stress have been reported previously in spider mites,^12^, ^17^, ^87^ and other arthropods,^78^, ^88^, ^89^ and may reflect differences in body size, energetic requirements, or exposure to risk during feeding and oviposition.^90^, ^91^ In spider mites, females are larger, more sedentary during feeding and oviposition, and nutritionally richer, making them more profitable and susceptible targets for predators.^92^ In contrast, males are smaller and can engage in defensive behaviors to reduce predation risk.^93^ Consequently, mothers under stress may therefore invest differently in daughters, who carry the direct burden of future egg production, or daughters may be more sensitive to the same maternal signals. The slowed development in daughters may be a conservative strategy, reducing their activity and visibility during vulnerable juvenile stages, but it also delays their entry into the reproductive pool, further inhibiting population growth.

### 4.4 Non‐consumptive stress suppresses population growth

Although reproductive acceleration in predation‐stress females of *T. ludeni* might provide a short‐term buffering at the individual level (Fig. 3), its population‐level consequences were strongly negative. As shown in Table 1, all life table parameters associated with population growth (*R₀*, *r*_*m*_, and *λ*) significantly declined with increasing predation stress, while doubling time (*Dt*) significantly increased, indicating slower population expansion. These results demonstrate that NCEs alone were sufficient to substantially slow population expansion, even in the absence of direct predation. Such findings agree with those of previous studies that fear‐mediated effects can rival or exceed consumptive effects in regulating prey populations.^2^, ^7^, ^8^

The discrepancy between individual‐level reproductive acceleration and population‐level decline in *T. ludeni* highlights the cumulative nature of physiological stress. The reduced longevity (Fig. 1), constrained fecundity (Fig. 2), and impaired offspring performance (Figs. 4 and 5) collectively limit the long‐term growth potential of *T. ludeni* populations (Table 1). Similar patterns have been reported in other predator‐spider mite systems, where predator cues suppressed population growth across multiple generations.^12^, ^87^ These results reinforce the conclusion that NCEs contribute to pest suppression not by directly eliminating individuals, but by altering life‐history trajectories in ways that inhibit the population growth rate.

### 4.5 Predator eggs expand functional scopes of biological control through fear‐mediated population suppression

We demonstrate that predator eggs of *P. persimilis*, as a precursor to juvenile predators, created a sustained background risk that could suppress pest populations before predators reach high densities. Similar fear‐mediated suppression has been documented in other agricultural systems, where predator cues reduced herbivore feeding, reproduction, and population growth.^13^, ^15^ Thus, predators may exert dual control, i.e., direct predation by active stages and fear‐mediated suppression via cues from eggs and other non‐feeding stages. These NCEs effectively broaden the functional role of predators across their own life cycle, leading to more efficient and sustainable pest management.^7^, ^9^ For *Tetranychus* species, the notorious pests with rapid acaricide resistance development,^36^, ^46^, ^47^, ^94^ incorporating NCEs into biological control strategies may enhance pest population suppression while reducing reliance on chemical inputs. Recognizing predator eggs as functional contributors to pest control broadens the scope of predator efficacy and underscores the importance of predator establishment, persistence, and reproductive activity in agroecosystems. Future strategies could consider early or inoculative releases of predators to establish this ‘landscape of fear’ before pest populations escalate, optimizing the synergy between consumptive and non‐consumptive effects for more resilient and effective biological control.

## 5 CONCLUSION

Predation stress induced by *P. persimilis* eggs generates strong, cue intensity‐dependent NCEs on spider mite *T. ludeni* development, survival, and reproduction, thereby altering prey life histories. Our results show that *T. ludeni* females can perceive and assess future predation risk and proactively adjust life‐history strategies. These responses involve shifts in resource allocation among growth, maintenance, and reproduction, which accelerate the initial reproduction but impose substantial fitness costs, including reduced survivorship and fecundity, highlighting the importance of physiological stress pathways in mediating non‐lethal NCEs. Predation stress also elicited sex‐specific responses, with female offspring exhibiting greater sensitivity to prolong development than males. This pattern likely reflects female‐biased body size, higher energetic demands associated with egg production, and increased exposure to risk during sedentary feeding and oviposition. Therefore, incorporating early NCEs and predation stress intensity into assessing the potential of predators in suppressing prey populations fills a critical gap in spider mite ecology and highlights the ecological significance of fear‐mediated effects in agricultural systems. Accounting for these NCEs will improve predictions of pest population dynamics and enhance biological control strategies. Nevertheless, although the predator egg densities used in this study were designed to create a controlled gradient of predation risk, their direct correspondence to field densities remains to be quantified. Future studies should link natural predator oviposition patterns with prey responses under greenhouse or field conditions to evaluate the applied significance of these non‐consumptive effects.

## CONFLICT OF INTEREST

The authors have no conflicts of interest.

## Data Availability

The data that support the findings of this study are available from the corresponding author upon reasonable request.

## References

[1] Peacor SD and Werner EE , The contribution of trait‐mediated indirect effects to the net effects of a predator. Proc Natl Acad Sci U S A 98:3904–3908 (2001). 10.1073/pnas.071061998. 11259674 PMC31151

[2] Preisser EL , Bolnick DI and Benard MF , Scared to death? The effects of intimidation and consumption in predator–prey interactions. Ecology 86:501–509 (2005). 10.1890/04-0719.

[3] Adamo SA , The stress response and immune system share, borrow, and reconfigure their physiological network elements: evidence from the insects. Horm Behav 88:25–30 (2017). 10.1016/j.yhbeh.2016.10.003. 27746212

[4] Hermann SL and Landis DA , Scaling up our understanding of non‐consumptive effects in insect systems. Curr Opin Insect Sci 20:54–60 (2017). 10.1016/j.cois.2017.03.010. 28602236

[5] Werner EE and Peacor SD , A review of trait‐mediated indirect interactions in ecological communities. Ecology 84:1083–1100 (2003). 10.1890/0012-9658(2003)084[1083:AROTII]2.0.CO;2.

[6] Clinchy M , Sheriff MJ and Zanette LY , Predator‐induced stress and the ecology of fear. Funct Ecol 27:56–65 (2013). 10.1111/1365-2435.12007.

[7] Ingerslew KS and Finke DL , Non‐consumptive effects stabilize herbivore control over multiple generations. PLoS One 15:e0241870 (2020). 10.1371/journal.pone.0241870. 33170896 PMC7654827

[8] Mutz J , Thaler JS , Ugine TA , Inouye BD and Underwood N , Predator densities alter the influence of non‐consumptive effects on the population dynamics of an agricultural pest. Ecol Entomol 49:306–318 (2024). 10.1111/een.13303.

[9] Preisser EL and Bolnick DI , The many faces of fear: comparing the pathways and impacts of nonconsumptive predator effects on prey populations. PLoS One 3:e2465 (2008). 10.1371/journal.pone.0002465. 18560575 PMC2409076

[10] Culshaw‐Maurer M , Sih A and Rosenheim JA , Bugs scaring bugs: enemy‐risk effects in biological control systems. Ecol Lett 23:1693–1714 (2020). 10.1111/ele.13601. 32902103 PMC7692946

[11] Stenberg JA , Sundh I , Becher PG , Björkman C , Dubey M , Egan PA *et al*., When is it biological control? A framework of definitions, mechanisms, and classifications. J Pest Sci 94:665–676 (2021). 10.1007/s10340-021-01354-7.

[12] Ristyadi D , He XZ and Wang Q , Predator‐ and killed prey‐induced fears bear significant cost to an invasive spider mite: implications in pest management. Pest Manag Sci 78:5456–5462 (2022). 10.1002/ps.7168. 36057852 PMC9826069

[13] Batabyal A , Predator‐prey systems as models for integrative research in biology: the value of a non‐consumptive effects framework. J Exp Biol 226:jeb245851 (2023). 10.1242/jeb.245851. 37772622

[14] Hawlena D and Schmitz OJ , Physiological stress as a fundamental mechanism linking predation to ecosystem functioning. Am Nat 176:537–556 (2010). 10.1086/656495. 20846014

[15] Thaler JS , McArt SH and Kaplan I , Compensatory mechanisms for ameliorating the fundamental trade‐off between predator avoidance and foraging. Proc Natl Acad Sci U S A 109:12075–12080 (2012). 10.1073/pnas.1208070109. 22778426 PMC3409718

[16] Dias CR , Bernardo AMG , Mencalha J , Freitas CWC , Sarmento RA , Pallini A *et al*., Antipredator behaviours of a spider mite in response to cues of dangerous and harmless predators. Exp Appl Acarol 69:263–276 (2016). 10.1007/s10493-016-0042-5. 27067101 PMC4891363

[17] Wei X and Zhang ZQ , Level‐dependent effects of predation stress on prey development, lifespan and reproduction in mites. Biogerontology 23:515–527 (2022). 10.1007/s10522-022-09980-z. 35879518 PMC9388410

[18] Lima SL and Dill LM , Behavioral decisions made under the risk of predation: a review and prospectus. Can J Zool 68:619–640 (1990). 10.1139/z90-092.

[19] Kats LB and Dill LM , The scent of death: chemosensory assessment of predation risk by prey animals. Ecoscience 5:361–394 (1998). 10.1080/11956860.1998.11682468.

[20] Stankowich T and Blumstein DT , Fear in animals: a meta‐analysis and review of risk assessment. Proc R Soc B 272:2627–2634 (2005). 10.1098/rspb.2005.3251. PMC1559976 16321785

[21] Ferrari MC , Wisenden BD and Chivers DP , Chemical ecology of predator–prey interactions in aquatic ecosystems: a review and prospectus. Can J Zool 88:698–724 (2010). 10.1139/Z10-029.

[22] Weissburg M , Smee DL and Ferner MC , The sensory ecology of nonconsumptive predator effects. Am Nat 184:141–157 (2014). 10.1086/676644. 25058276

[23] Ferrari MC , Messier F and Chivers DP , The nose knows: minnows determine predator proximity and density through detection of predator odours. Anim Behav 72:927–932 (2006). 10.1016/j.anbehav.2006.03.001.

[24] Dicke M and Grostal P , Chemical detection of natural enemies by arthropods: an ecological perspective. Annu Rev Ecol Syst 32:1–23 (2001). 10.1146/annurev.ecolsys.32.081501.113951.

[25] Hermann SL and Thaler JS , Prey perception of predation risk: volatile chemical cues mediate non‐consumptive effects of a predator on a herbivorous insect. Oecologia 176:669–676 (2014). 10.1007/s00442-014-3069-5. 25234373

[26] Poulin RX , Lavoie S , Siegel K , Gaul DA , Weissburg MJ and Kubanek J , Chemical encoding of risk perception and predator detection among estuarine invertebrates. Proc Natl Acad Sci U S A 115:662–667 (2018). 10.1073/pnas.1713901115. 29311305 PMC5789921

[27] Grunseich JM , Aguirre NM , Thompson MN , Ali JG and Helms AM , Chemical cues from entomopathogenic nematodes vary across three species with different foraging strategies, triggering different behavioral responses in prey and competitors. J Chem Ecol 47:822–833 (2021). 10.1007/s10886-021-01304-8. 34415500 PMC8613145

[28] McCoy MW , Touchon JC , Landberg T , Warkentin KM and Vonesh JR , Prey responses to predator chemical cues: disentangling the importance of the number and biomass of prey consumed. PLoS One 7:e47495 (2012). 10.1371/journal.pone.0047495. 23082171 PMC3474732

[29] Adamo SA and McKee R , Differential effects of predator cues versus activation of fight‐or‐flight behaviour on reproduction in the cricket *Gryllus texensis* . Anim Behav 134:1–8 (2017). 10.1016/j.anbehav.2017.09.027.

[30] Wei X and Zhang ZQ , Dosage‐dependent and prey stage‐specific non‐consumptive effects of predators on prey: interactions between *Neoseiulus cucumeris* and *Tyrophagus putrescentiae* . Zoosymposia 22:117–120 (2022). 10.11646/zoosymposia.22.1.72.

[31] Stevens M , Sensory ecology, information, and decision‐making, in Sensory Ecology, Behaviour, and Evolution, ed. by Stevens M . Oxford University Press, Oxford, UK, pp. 1–17 (2013). 10.1093/acprof:oso/9780199601776.001.0001.

[32] Narimanov N , Heuschele JM , Entling MH , Menzel F and Mestre L , Differential effects of ephemeral and stable predator chemical cues on spider antipredator behaviour. J Chem Ecol 50:714–724 (2024). 10.1007/s10886-024-01543-5. 39305439 PMC11543770

[33] Janssens L and Stoks R , Predation risk causes oxidative damage in prey. Biol Lett 9:20130350 (2013). 10.1098/rsbl.2013.0350. 23760170 PMC3730648

[34] Sitvarin M , Breen K and Rypstra A , Predator cues have contrasting effects on lifespan of *Pardosa malvina* (Araneae: Lycosidae). J Arachnol 43:107–110 (2015). 10.1636/J14-48.1.

[35] Helle W and Sabelis MW , Spider mites: their biology, in Natural Enemies and Control. Elsevier Science Publishers B.V., Amsterdam (1985).

[36] Van Leeuwen T , Vontas J , Tsagkarakou A , Dermauw W and Tirry L , Acaricide resistance mechanisms in the two‐spotted spider mite *Tetranychus urticae* and other important Acari: a review. Insect Biochem Mol Biol 40:563–572 (2010). 10.1016/j.ibmb.2010.05.008. 20685616

[37] Boubou A , Migeon A , Roderick GK and Navajas M , Recent emergence and worldwide spread of the red tomato spider mite, *Tetranychus evansi*: genetic variation and multiple cryptic invasions. Biol Invasions 13:81–92 (2011). 10.1007/s10530-010-9791-y.

[38] Jakubowska M , Dobosz R , Zawada D and Kowalska J , A review of crop protection methods against the two‐spotted spider mite—*Tetranychus urticae* Koch (Acari: Tetranychidae)—with special reference to alternative methods. Agri 12:898 (2022). 10.3390/agriculture12070898.

[39] Zhang Z‐Q , Taxonomy of *Tetranychus ludeni* (Acari: Tetranychidae) in New Zealand and its ecology on *Sechium edule* . N Z Entomol 25:27–34 (2002). 10.1080/00779962.2002.9722091.

[40] Marić I , Međo I and Marčić D , The dark‐red spider mite, *Tetranychus ludeni* Zacher (Acari: Tetranychidae) – a new pest in *Serbian acarofauna* . Pestic Fitomed 37:85–93 (2022). 10.2298/PIF2203085M.

[41] Godinho DP , Janssen A , Dias T , Cruz C and Magalhães S , Down‐regulation of plant defence in a resident spider mite species and its effect upon con‐ and heterospecifics. Oecologia 180:161–167 (2016). 10.1007/s00442-015-3434-z. 26369779

[42] Reichert MB , Schneider JR , Wurlitzer WB and Ferla NJ , Impacts of cultivar and management practices on the diversity and population dynamics of mites in soybean crops. Exp Appl Acarol 92:41–59 (2024). 10.1007/s10493-023-00862-8. 38036759

[43] Wurlitzer WB , Schneider JR , Silveira JAG , de Almeida Oliveira MG , Labudda M , Chavarria G *et al*., *Tetranychus ludeni* (Acari: Tetranychidae) infestation triggers a spatiotemporal redox response dependent on soybean genotypes. Planta 260:130 (2024). 10.1007/s00425-024-04566-0. 39487857

[44] Escudero LA and Ferragut F , Life‐history of predatory mites *Neoseiulus californicus* and *Phytoseiulus persimilis* (Acari: Phytoseiidae) on four spider mite species as prey, with special reference to *Tetranychus evansi* (Acari: Tetranychidae). Biol Control 32:378–384 (2005). 10.1016/j.biocontrol.2004.12.010.

[45] Reis PR , Predatory mites for biological control of phytophagous mites, in Natural Enemies of Insect Pests in Neotropical Agroecosystems, ed. by Souza B , Vázquez L and Marucci R . Springer, Cham, pp. 189–198 (2019). 10.1007/978-3-030-24733-1_16.

[46] Dağlı F and Tunç İ , Dicofol resistance in *Tetranychus cinnabarinus*: resistance and stability of resistance in populations from Antalya, Turkey. Pest Manag Sci 57:609–614 (2001). 10.1002/ps.334. 11464792

[47] Bi JL , Niu ZM , Yu L and Toscano NC , Resistance status of the carmine spider mite, *Tetranychus cinnabarinus*, and the two‐spotted spider mite, *Tetranychus urticae*, to selected acaricides on strawberries. Insect Sci 23:88–93 (2016). 10.1111/1744-7917.12190. 25409919

[48] Hoffmeister TS and Roitberg BD , Counterespionage in an insect herbivore‐parasitoid system. Naturwissenschaften 84:117–119 (1997). 10.1007/s001140050358.

[49] Grostal P and Dicke M , Direct and indirect cues of predation risk influence behavior and reproduction of prey: a case for acarine interactions. Behav Ecol 10:422–427 (1999). 10.1093/beheco/10.4.422.

[50] Grostal P and Dicke M , Recognising one's enemies: a functional approach to risk assessment by prey. Behav Ecol Sociobiol 47:258–264 (2000). 10.1007/s002650050663.

[51] Pallini A , Janssen A and Sabelis MW , Spider mites avoid plants with predators. Exp Appl Acarol 23:803–815 (1999). 10.1023/A:1006266232714.

[52] Dittmann L and Schausberger P , Adaptive aggregation by spider mites under predation risk. Sci Rep 7:10609 (2017). 10.1038/s41598-017-10819-8. 28878255 PMC5587541

[53] Shimoda T , Shinkaji N and Amano H , Prey stage preference and feeding behaviour of *Oligota kashmirica benefica* (Coleoptera: Staphylinidae), an insect predator of the spider mite *Tetranychus urticae* (Acari: Tetranychidae). Exp Appl Acarol 21:665–675 (1997). 10.1007/BF02803509.

[54] Gotoh T , Nozawa M and Yamaguchi K , Prey consumption and functional response of three acarophagous species to eggs of the two‐spotted spider mite in the laboratory. Appl Entomol Zool 39:97–105 (2004). 10.1303/aez.2004.97.

[55] Oliveira H , Janssen A , Pallini A , Venzon M , Fadini M and Duarte V , A phytoseiid predator from the tropics as potential biological control agent for the spider mite *Tetranychus urticae* Koch (Acari: Tetranychidae). Biol Control 42:105–109 (2007). 10.1016/j.biocontrol.2007.04.011.

[56] Ali MP , Naif AA and Huang D , Prey consumption and functional response of a phytoseiid predator, *Neoseiulus womersleyi*, feeding on spider mite, Tetranychus macfarlanei. J Insect Sci 11:167 (2011). 10.1093/jis/11.1.167.

[57] Xiao Y , Osborne LS , Chen J and McKenzie CL , Functional responses and prey‐stage preferences of a predatory gall midge and two predacious mites with twospotted spider mites, *Tetranychus urticae*, as host. J Insect Sci 13:8 (2013). 10.1673/031.013.0801. 23879370 PMC3735104

[58] Krey KL , Blubaugh CK , Chapman EG , Lynch CA , Snyder GB , Jensen AS *et al*., Generalist predators consume spider mites despite the presence of alternative prey. Biol Control 115:157–164 (2017). 10.1016/j.biocontrol.2017.10.007.

[59] Simkhada R , Kundun J , Sofkova‐Bobcheva S and He XZ , Stronger antipredatory vigilance of prey to olfactory cues from injured vulnerable conspecifics. Ecol Evol 15:e72257 (2025). 10.1002/ece3.72257. 41059357 PMC12498088

[60] Gyuris E , Szep E , Kontschán J , Hettyey A and Toth Z , Behavioural responses of two‐spotted spider mites induced by predator‐borne and prey‐borne cues. Behav Processes 144:100–106 (2017). 10.1016/j.beproc.2017.09.002. 28882653

[61] Murase A , Fujita K and Yano S , Behavioural flexibility in spider mites: oviposition site shifts based on past and present stimuli from conspecifics and predators. R Soc Open Sci 4:170328 (2017). 10.1098/rsos.170328. 28791161 PMC5541556

[62] Walzer A and Schausberger P , Integration of multiple intraguild predator cues for oviposition decisions by a predatory mite. Anim Behav 84:1411–1417 (2012). 10.1016/j.anbehav.2012.09.006. 23264692 PMC3518781

[63] Walzer A and Schausberger P , Integration of multiple cues allows threat‐sensitive anti‐intraguild predator responses in predatory mites. Behaviour 150:115–132 (2013). 10.1163/1568539X-00003040. 23750040 PMC3672986

[64] Jervis MA , Copland MJW , Shameer KS and Harvey JA , The life‐cycle, in Jervis's Insects as Natural Enemies: Practical Perspectives, ed. by Hardy ICW and Wajnberg E . Springer, Cham, pp. 105–232 (2023). 10.1007/978-3-031-23880-2_2.

[65] Huang Y‐B and Chi H , Assessing the application of the jackknife and bootstrap techniques to the estimation of the variability of the net reproductive rate and gross reproductive rate: a case study in *Bactrocera cucurbitae* (Coquillett) (Diptera: Tephritidae). J Agric For 61:37–46 (2012). 10.30089/JAF.201203.0003.

[66] Yu L‐Y , Chen Z‐Z , Zheng F‐Q , Shi A‐J , Guo T‐T , Yeh B‐H *et al*., Demographic analysis, a comparison of the jackknife and bootstrap methods, and predation projection: a case study of *Chrysopa pallens* (Neuroptera: Chrysopidae). J Econ Entomol 106:1–9 (2013). 10.1603/EC12200. 23448008

[67] Archontoulis SV and Miguez FE , Nonlinear regression models and applications in agricultural research. Agron J 107:786–798 (2015). 10.2134/agronj2012.0506.

[68] Julious SA , Using confidence intervals around individual means to assess statistical significance between two means. Pharm Stat 3:217–222 (2004). 10.1002/pst.126.

[69] Azandémè‐Hounmalon GY , Torto B , Fiaboe KKM , Subramanian S , Kreiter S and Martin T , Visual, vibratory and olfactory cues affect interactions between the red spider mite *Tetranychus evansi* and its predator *Phytoseiulus longipes* . J Pest Sci 89:137–152 (2016). 10.1007/s10340-015-0682-y.

[70] Murase A and Fujita K , Predator experience changes spider mites' habitat choice even without current threat. Sci Rep 8:8388 (2018). 10.1038/s41598-018-26757-y. 29849059 PMC5976667

[71] Bowler DE , Yano S and Amano H , The non‐consumptive effects of a predator on spider mites depend on predator density. J Zool 289:52–59 (2013). 10.1111/j.1469-7998.2012.00961.x.

[72] Duffield KR , Bowers EK , Sakaluk SK and Sadd BM , A dynamic threshold model for terminal investment. Behav Ecol Sociobiol 71:1–17 (2017). 10.1007/s00265-017-2416-z. PMC6039117 30002566

[73] Farchmin PA , Eggert A‐K , Duffield KR and Sakaluk SK , Dynamic terminal investment in male burying beetles. Anim Behav 163:1–7 (2020). 10.1016/j.anbehav.2020.02.015.

[74] Sheriff MJ , Peacor SD , Hawlena D and Thaker M , Non‐consumptive predator effects on prey population size: a dearth of evidence. J Anim Ecol 89:1302–1316 (2020). 10.1111/1365-2656.13213. 32215909

[75] Schulz NKE , Stewart CM and Tate AT , Female investment in terminal reproduction or somatic maintenance depends on infection dose. Ecol Entomol 48:714–724 (2023). 10.1111/een.13268.

[76] Choh Y , Uefune M and Takabayashi J , Predation‐related odours reduce oviposition in a herbivorous mite. Exp Appl Acarol 50:1–8 (2010). 10.1007/s10493-009-9277-8. 19526199

[77] Luong LT , Horn CJ and Brophy T , Mitey costly: energetic costs of parasite avoidance and infection. Physiol Biochem Zool 90:471–477 (2017). 10.1086/691704. 28414262

[78] Liu X , Wen J , Geng X , Xiao L , Zou Y , Shan Z *et al*., The impact of predation risks on the development and fecundity of *Bactrocera dorsalis* Hendel. Insects 15:322 (2024). 10.3390/insects15050322. 38786878 PMC11122621

[79] Wineland SM , Kistner EJ and Joern A , Non‐consumptive interactions between grasshoppers (Orthoptera: Acrididae) and wolf spiders (Lycosidae) produce trophic cascades in an old‐field ecosystem. J Orthoptera Res 24:41–46 (2015). 10.1665/034.024.0101.

[80] Des Roches S , Robinson RR , Kinnison MT and Palkovacs EP , The legacy of predator threat shapes prey foraging behaviour. Oecologia 198:79–89 (2022). 10.1007/s00442-021-05073-9. 34817645

[81] Mezheritskiy MI , Vorontsov DD , Dyakonova VE and Zakharov IS , Behavioral functions of octopamine in adult insects under stressful conditions. J Gen Biol 85:3–16 (2024). 10.31857/S0044459624010015.

[82] Yang H , Du J , Wang L , Zhu P , Li D , Huang J *et al*., Predation risk effects of *Harmonia axyridis* on the development and fecundity of *Periphyllus koelreuteriae* . Insects 16:695 (2025). 10.3390/insects16070695. 40725325 PMC12295122

[83] Freinschlag J and Schausberger P , Predation risk‐mediated maternal effects in the two‐spotted spider mite, Tetranychus urticae. Exp Appl Acarol 69:35–47 (2016). 10.1007/s10493-016-0014-9. 26923463

[84] McGhee KE , Barbosa AJ , Bissell K , Darby NA and Foshee S , Maternal stress during pregnancy affects activity, exploration and potential dispersal of daughters in an invasive fish. Anim Behav 171:41–50 (2021). 10.1016/j.anbehav.2020.11.003.

[85] Sharda S , Zuest T , Erb M and Taborsky B , Predator‐induced maternal effects determine adaptive antipredator behaviors via egg composition. Proc Natl Acad Sci U S A 118:e2017063118 (2021). 10.1073/pnas.2017063118. 34507981 PMC8449421

[86] McGhee KE , Pintor LM , Suhr EL and Bell AM , Maternal exposure to predation risk decreases offspring antipredator behaviour and survival in threespined stickleback. Funct Ecol 26:932–940 (2012). 10.1111/j.1365-2435.2012.02008.x. 22962510 PMC3434968

[87] Li GY and Zhang ZQ , Development, lifespan and reproduction of spider mites exposed to predator‐induced stress across generations. Biogerontology 20:871–882 (2019). 10.1007/s10522-019-09835-0. 31535231

[88] Wormington JD and Juliano SA , Hunger‐dependent and sex‐specific antipredator behaviour of larvae of a size‐dimorphic mosquito. Ecol Entomol 39:548–555 (2014). 10.1111/een.12129. 25309025 PMC4190168

[89] Donelan SC and Trussell GC , Sex‐specific differences in the response of prey to predation risk. Funct Ecol 34:1235–1243 (2020). 10.1111/1365-2435.13569.

[90] Wei X , Liu J and Zhang Z‐Q , Predation stress experienced as immature mites extends their lifespan. Biogerontology 24:67–79 (2023). 10.1007/s10522-022-09990-x. 36085209 PMC9845153

[91] Wei X , Li G and Zhang Z‐Q , Prey life stages modulate effects of predation stress on prey lifespan, development, and reproduction in mites. Insect Sci 30:844–856 (2023). 10.1111/1744-7917.13124. 36271685

[92] Li GY and Zhang ZQ , Sex dimorphism of life‐history traits and their response to environmental factors in spider mites. Exp Appl Acarol 84:497–527 (2021). 10.1007/s10493-021-00632-4. 34125333

[93] Mortezapour K , Golpayegani AZ , Saboori A and Mohammadi H , *Tetranychus urticae* (Acari: Tetranychidae) male defensive behavior against *Phytoseiulus persimilis* (Acari: Phytoseiidae). Persian J Acarol 11:671–680 (2022). 10.22073/pja.v11i4.70423.

[94] Nyoni BN , Gorman K , Mzilahowa T , Williamson MS , Navajas M , Field LM *et al*., Pyrethroid resistance in the tomato red spider mite, *Tetranychus evansi*, is associated with mutation of the para‐type sodium channel. Pest Manag Sci 67:891–897 (2011). 10.1002/ps.2145. 21432985

